# AI-Driven Decision Support for Early Detection of Cardiac Events: Unveiling Patterns and Predicting Myocardial Ischemia

**DOI:** 10.3390/jpm13091421

**Published:** 2023-09-21

**Authors:** Luís B. Elvas, Miguel Nunes, Joao C. Ferreira, Miguel Sales Dias, Luís Brás Rosário

**Affiliations:** 1 ISTAR, Instituto Universitário de Lisboa (ISCTE-IUL), 1649-026 Lisbon, Portugal; miguel_bonacho@iscte-iul.pt (M.N.); jcafa@iscte-iul.pt (J.C.F.); miguel.dias@iscte-iul.pt (M.S.D.); 2 Inov Inesc Inovação—Instituto de Novas Tecnologias, 1000-029 Lisbon, Portugal; 3 Faculty of Medicine, Lisbon University, Hospital Santa Maria/CHULN, CCUL, 1649-028 Lisbon, Portugal; lsrosario@medicina.ulisboa.pt

**Keywords:** cardiovascular diseases, myocardial infarction, pulmonary thromboembolism, aortic stenosis, stenosis cardiology, exploratory data analysis, artificial intelligence, machine learning, data mining, prediction

## Abstract

Cardiovascular diseases (CVDs) account for a significant portion of global mortality, emphasizing the need for effective strategies. This study focuses on myocardial infarction, pulmonary thromboembolism, and aortic stenosis, aiming to empower medical practitioners with tools for informed decision making and timely interventions. Drawing from data at Hospital Santa Maria, our approach combines exploratory data analysis (EDA) and predictive machine learning (ML) models, guided by the Cross-Industry Standard Process for Data Mining (CRISP-DM) methodology. EDA reveals intricate patterns and relationships specific to cardiovascular diseases. ML models achieve accuracies above 80%, providing a 13 min window to predict myocardial ischemia incidents and intervene proactively. This paper presents a Proof of Concept for real-time data and predictive capabilities in enhancing medical strategies.

## 1. Introduction

Cardiovascular diseases are the leading cause of mortality worldwide. In 2020, cardiovascular diseases (CVDs) accounted for 17.9 million deaths, or 32% of all global deaths [1,2]. CVDs are also a leading cause of hospitalization and disability. Addressing these complexities requires innovative approaches that empower medical practitioners to make informed decisions, leading to improved patient outcomes and more effective healthcare strategies [2]. Notably, a study by Oxford Population Health’s Health Economics Research Centre unveiled that in 2021, cardiovascular diseases incurred a cost of EUR 282 billion in the European Union (EU) economy [3]. This economic burden emphasizes the urgent need for innovative approaches that enhance medical decisions and healthcare strategies, ultimately improving patient outcomes.

In modern medical practice, physicians are confronted with intricate clinical scenarios that demand timely and data-driven interventions [4]. In the realm of Intensive Care Units, the ability to harness comprehensive patient data for insightful decisions has the potential to dramatically impact patient care and enhance healthcare quality [5]. In the current medical practice, patients have several physiologic parameters monitored—e.g., Heart Rate, Blood Pressure, Oxymetry, Body Temperature—that raise alarms when pre specified thresholds are crossed, which prompts diagnostic or therapeutic interventions. In this sense, patient care is triggered after the fact, as if a car driver were driving looking at the rear mirror. This is in contrast with other sciences and work practices, for example, Meteorology, where prediction drives Agriculture or Navigation decisions, based on data-driven models.

The intersection of medical technology and data science has opened new avenues for tackling disease prediction. Machine learning, a subset of artificial intelligence, promises to unravel intricate patterns within vast datasets. Its modeling techniques, capable of extracting meaningful insights from complex clinical information, coupled with its predictive prowess, could reshape how cardiac diseases are diagnosed, treated, and even forecasted and prevented.

Machine learning techniques (ML) offer a transformative paradigm in cardiovascular healthcare, enabling the integration of diverse data sources to unveil hidden correlations, prognostic markers, and emerging risk factors [6]. This technology has the potential to empower clinicians with predictive tools that can anticipate adverse cardiac events, enabling early interventions and personalized treatment strategies. Early diagnosis and intervention are essential for improving the outcomes of patients with CVDs [7]. This is where machine learning can play a valuable role that can be used to analyze large amounts of data and identify patterns that would be difficult to detect by human experts [8]. ML has been shown to be effective in predicting CVDs, even in patients who have no symptoms [9]. The precision of these models holds the potential to improve patient care, reduce hospitalizations, and mitigate the long-term impact of cardiac diseases [9].

Our approach showcases the capabilities unlocked through structured health database analysis from a real-world problem. In our quest to advance cardiovascular healthcare, our study adopts an innovative approach that underscores both privacy and collaboration. Importantly, these data were homomorphically encrypted to uphold privacy and confidentiality standards. The application of Data Sharing Agreements (DSAs) ensures responsible and compliant data sharing practices, safeguarding patient information [10].

Our commitment to enhance cardiovascular healthcare is mapped in a central research question: “How can fusion of Exploratory Data Analysis (EDA) techniques and predictive Machine Learning models assist medical staff in accurate clinical decision-making, and facilitate timely medical interventions of a preventive nature?”. This pivotal question guides our exploration into harnessing data-driven methodologies to drive innovative solutions in the context of cardiac care.

The core objectives of our study are two-fold, aligning seamlessly with the holistic nature of our research question. Firstly, we endeavor to unravel intricate patterns within the multi-syndrome dataset from Hospital Santa Maria through meticulous EDA. This analytical journey offers insights into disease-specific trends, risk factors, and underlying relationships, thereby equipping medical professionals with a deeper understanding of cardiovascular diseases for enhanced diagnosis, prognosis, and treatment strategies. Secondly, we are dedicated to harnessing the predictive power of machine learning models to anticipate myocardial ischemia. By utilizing the knowledge gained from our exploratory analysis, we aim to develop intelligent predictive models capable of forecasting cardiac incidents with a high degree of accuracy. This predictive capability has the potential to empower healthcare practitioners to implement preemptive measures, enabling timely interventions that significantly impact patient outcomes.

Our main objective, as directed by physicians, was to explore and extract knowledge from patient data related to three specific diseases: myocardial infarction, pulmonary thromboembolism, and aortic stenosis, because they serve as useful comparators for COVID-19 (coronavirus disease 2019), the newly emerged disease.

This paper illustrates the potential of AI-driven approaches to health data analysis. While we focus on myocardial infarction, pulmonary thromboembolism, and aortic stenosis for illustrative purposes, the underpinning principle is universally applicable, including for patients diagnosed with COVID-19. With structured, annotated and well-prepared data (including physiological data), these methodologies can be extended to address any other diseases, harnessing the power of data and technology to pioneer enhanced healthcare.

In summary, our paper charts a path towards addressing cardiovascular diseases by leveraging data analysis and predictive machine learning models. By harmonizing advanced technology with medical insights, we equip clinicians with the tools to aid them in making informed accurate decisions, pre-empt risk situations, and optimize patient care and clinical outcomes. In the rapidly advancing field of cardiovascular healthcare, our study is a significant contribution, providing data-driven evidence-based insights that have implications for improved patient outcomes.

## 2. State of the Art

In this section, we went through the existing body of knowledge in the realm of artificial intelligence (AI) applications within cardiovascular diseases. We followed the PRISMA methodology (Preferred Reporting Items for Systematic Reviews and Meta-Analysis) [11], not merely as a matter of convention, but to illuminate the path we have forged in pursuit of our research objectives. We recognize the potential for questions to arise regarding the integration of this comprehensive literature review into our broader study. Therefore, it is essential to clarify the rationale and significance of our approach.

The literature review within this study serves a dual role that is both foundational and contextual. We utilize it to identify research gaps and limitations that have guided the formulation of our research questions. Furthermore, it places our study within the broader landscape of AI-driven healthcare, offering readers a glimpse into the evolution and current state of the art. One of our primary objectives in conducting this literature review was to identify critical research gaps and limitations in existing studies. These gaps, as illuminated through our systematic review process, have played a pivotal role in shaping the specific research questions addressed in this article. Our intention is not to overshadow the primary focus of our research but to underscore the significance of our contributions by addressing unresolved questions in the field.

### 2.1. Search Strategy and Inclusion Criteria

Conducted in July 2023, this literature review focused solely on articles and reviews written in English, published in journals between 2018 and 2023, sourced from the Scopus and Web of Science Core Collection databases. We removed any duplicated articles to ensure data integrity.

To ensure clarity in our search, we constructed a comprehensive search query encompassing the concepts of “Machine Learning”, “Artificial Intelligence”, or “Data Mining” applied to the context of “Decision Support System”, “Data Analytics”, or “Data Analysis”. This search was specifically targeted at the population of “Hospital Data” or “Health Data”, with additional filtering based on “Cardiology” or “Cardiovascular Disease”. We ended up with the following query “(“Machine Learning” OR “Artificial Intelligence” OR “Data Mining”) AND (“Decision Support System” OR “Data Analytic” OR “Data Analysis”) AND (“Hospital Data” OR “Health Data”) AND (“Cardiology” OR “Cardiovascular Disease”)”.

### 2.2. Results

**Figure 1:**
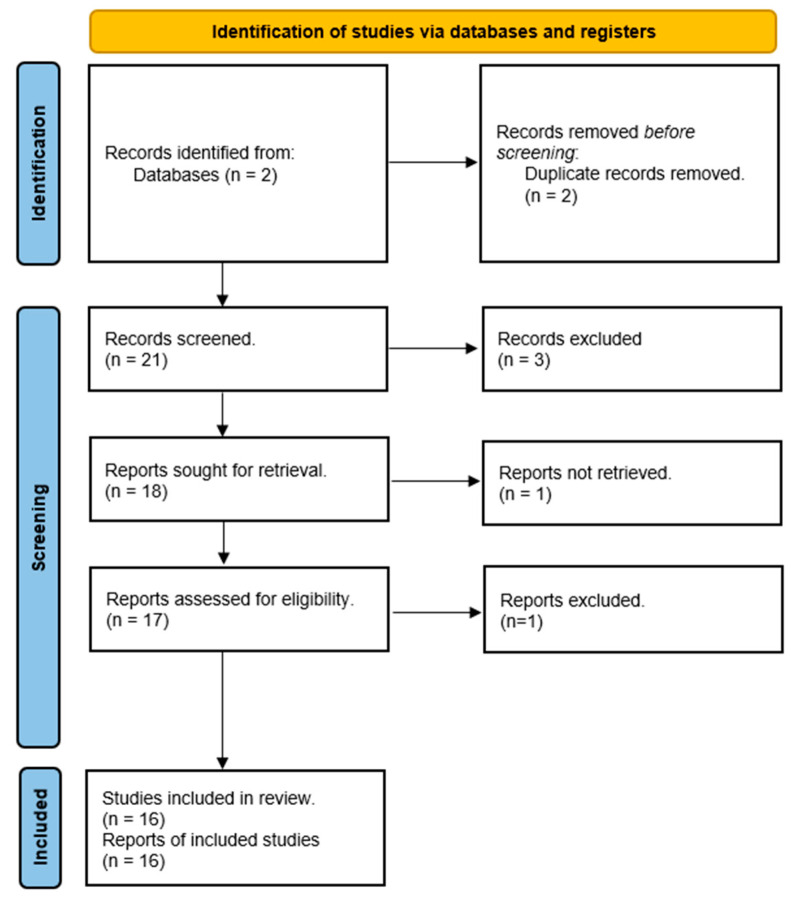
PRISMA workflow diagram.

The application of the mentioned query to the said Core Collection databases retrieved 21 papers. After the acquisition of such papers, we followed the PRISMA workflow, as depicted in Figure 1, illustrating our analysis of the reviewed articles.

Our goal was to investigate the application of Artificial Intelligence (AI) or machine learning in Health Data, with a specific focus on heart diseases. Throughout our literature review, we came across various topics related to this subject, and Table 1 summarizes the key themes found in each document. Without surprise, Heart Disease Prediction emerged as a prominent topic in this field, and the Internet of Things (IoT) also played a significant role in data acquisition, enabling further analysis. Additionally, the Risk Assessment of heart diseases or mortality was prevalent in the studies that were examined.

A more detailed review of each document is also presented next, where article [27] discusses the significance of data integration and introduces a diagnosis recommender system designed to assist physicians. In the same topic, ref. [26] presents a recommender-system solution that utilizes clustering techniques for each disease partition, including angina, non-cardiac chest pain, silent ischemia, and myocardial infarction.

Study [15] proposes the integration of Blockchain with AI to strengthen both technologies and create a novel solution that serves the objective of providing improvements in cardiovascular medicine.

Articles [12,13,14,21] use IoT sensors to capture data and then use data to predict and diagnose heart diseases with very promising results. Refs. [20,28] discuss the development of an optimized feature-selection algorithm designed to predict heart diseases at an early stage. Work [29] also discuss the development of a heart disease prediction model (on benchmarking datasets). Article [17] proposes the development of a machine learning algorithm to predict myocardial infarction diagnosis using electronic health record data readily available during Emergency Department assessments. Work [22] is a state of the art for using the Internet of Things with quantum dots in medicine. This integration offers advanced disease detection and personalized treatments through precise data collection. Healthcare benefits from the Artificial Intelligence-aided IoT, which securely transmits patient data for tailored solutions.

Work [18] discusses the establishment of early warning models to assess and prevent diseases such as stroke, heart failure, and renal failure. The authors of [16] utilize IoT biosensors in a machine learning-based risk-assessment approach. Ref. [19] focuses on predicting the mortality risk of patients during or shortly after cardiac surgery using machine learning techniques for cardiac risk assessment.

The authors of [23] present a comprehensive review that delves into the history of artificial intelligence in medicine, exploring its contemporary and future applications in adult and pediatric cardiology, with a focus on selected concentrations. The review also addresses the existing barriers to implementing these advanced technologies. Furthermore, the article concludes by discussing the notable advantages of having a recommender system in place. Such a system would not only enhance workflow efficiency but also provide physicians with more time to spend with their patients, leading to increased job satisfaction. As a result, patients are expected to experience improved satisfaction as they benefit from more face-to-face time with their physicians.

Globally, there is a concerted effort to maximize the advantages of artificial intelligence in medicine [23], aiming to assist physicians in achieving better performance and enhance patients’ experiences during hospitalization. However, we found a gap in the post-diagnosis phase. Following a patient’s hospitalization, they are connected to numerous medical devices and our study centers on the analysis of select data gathered from these devices, aiming to assist physicians in comprehending typical patient behavior post-diagnosis. Distinguishing itself from previous research, our primary emphasis lies in maximizing the utility of existing hospital medical devices and harnessing the resultant data in the post-diagnosis phase. Our objective is twofold: first, to identify patterns during the post-diagnosis phase that could aid physicians in better evaluating patients’ progress; second, we propose predicting potential cardiological complications that may arise during the hospitalization period and impact patients’ well-being.

## 3. Methodology

In the pursuit of our research objectives, we employed a systematic-approach CRISP-DM to guide the development of our study. Leveraging the comprehensive patient data from Hospital Santa Maria, we followed a structured methodology to uncover insights and develop predictive models. Our database, integral to this study, includes data from 512,764 patients and contains continuous clinical signals such as Temperature, Blood Oxygen Level (SpO2), Heart Rate, and Arterial Blood Pressure. This dataset, comprising 138 tables and occupying 75 gigabytes of data, was provided under the framework of the FCT project DSAIPA/AI/0122/2020 AIMHealth—Mobile Applications Based on Artificial Intelligence [11]. The availability of the database for research was approved by the Ethical Committee of the Faculty of Medicine of Lisbon, one of the project partners.

These patients were selected from the Medical Intensive Care Units of Hospital de Santa Maria, the largest Portuguese Public Hospital, located in Lisbon. They were already diagnosed with specific diseases, and we chose to identify or forecast myocardial ischemia, a daunting complication, in three specific diseases—Acute Myocardial Infarction, Pulmonary Thromboembolism, and Aortic Stenosis—that represent a spectrum of Cardiovascular Diseases (CVDs).

This approach was informed by industry-standard frameworks like the Cross-Industry Standard Process for Data Mining (CRISP-DM) [24]. The utilization of such methodologies ensures a rigorous and well-organized process, aligning with best practices while allowing us to focus on the medical significance and practical implications of our findings. Building upon a doctoral research initiative, Data Sharing Agreements were meticulously crafted and signed. The implementation of homomorphic encryption, initially explored in a previously published paper [10], imparted an additional layer of academic rigor and depth to our methodology.

The combination of systematic methodologies, comprehensive patient data, advanced ML techniques, and ethical considerations forms the robust foundation of our research, enabling us to pursue a deeper understanding of cardiovascular diseases and their predictive modeling.

By leveraging CRISP-DM, we aimed to develop models that are not only accurate but also meaningful for medical professionals navigating the complexities of cardiovascular healthcare. Within CRISP-DM, we divided our efforts into two key areas: (1) exploratory data analysis (EDA) and (2) machine learning (ML) predictive models. This division allowed us not only to conduct a comprehensive evaluation of the data and gain a more thorough understanding of each disease, but also to structure this study effectively.

### 3.1. Exploratory Data Analysis

In Section 4 of our study, we conducted EDA for decision support purposes, during which we analyzed each disease individually. This phase of our methodology aimed to uncover critical insights into the progression of myocardial ischemia within the context of three specific diseases: Acute Myocardial Infarction, Pulmonary Thromboembolism, and Aortic Stenosis. By thoroughly examining the data through EDA, we laid the foundation for our subsequent ML modeling efforts.

### 3.2. Machine Learning Predictive Models

Section 5 of our study marked the application of ML models to the diseases under study. This phase involved the implementation and evaluation of predictive models to identify or forecast myocardial ischemia within the specified diseases. By harnessing the power of advanced ML techniques, we sought to provide clinicians with valuable tools for making informed, accurate decisions, preempting risk situations, and optimizing patient care and clinical outcomes in the rapidly advancing field of cardiovascular healthcare.

## 4. Exploratory Data Analysis for Decision Support

With a firm commitment to elevating patient outcomes and enhancing medical strategies, this chapter embarks on a journey through the vast expanse of patient data collected from Hospital Santa Maria. By employing exploratory data analysis (EDA) techniques, we uncover hidden relationships, correlations, and trends that have the potential to redefine clinical decision making in cardiovascular healthcare. This expedition seeks to reveal nuanced intricacies that can significantly shape the course of patient care.

During the EDA phase, our primary objective was to unveil insights and hidden patterns within the data that hold the promise of aiding in early treatment or risk assessment. We concentrated on identifying patient profiles and recurrent patterns in frequently measured physiological data, along with examination results. This phase forms the bedrock of our research, providing a comprehensive understanding of disease-specific trends and risk factors that underpin the subsequent predictive models.

Our primary focus was on a table called RT_Data, which contains real-time data collected during patients’ hospital stays. This valuable table encompasses physiological data, vital signs, and information gathered from medical devices. Among all the variables, a few were selected by physicians to study their behavior and examine if there are any relevant patterns for each disease. It is crucial to emphasize that while numerous columns were available, our physicians’ colleagues meticulously handpicked the most pertinent ones for our disease study. The most commonly recorded physiological variables, Heart Rate, Respiratory Rate, Arterial systolic Blood Pressure, Arterial Diastolic Blood Pressure and Mean Arterial Pressure, were chosen as they reflect the momentaneous function of the cardiovascular and respiratory systems and their reflex regulation (see Table 2).

Additionally, we conducted a thorough examination of the diagnostic table (see Table 3), which played a crucial role in our analysis. This table provided essential details regarding the prescribed diagnoses for each patient, allowing us to filter and concentrate specifically on the diagnoses corresponding to the selected diseases: myocardial infarction, pulmonary thromboembolism, and aortic stenosis.

To construct comprehensive patient profiling, we gathered additional information from the patients table in Table 4, and also from the patient’s admission table in Table 5. The patient’s table enabled us to collect information about individual characteristics, while the patient’s admission table provided details about patient’s weight, height, and the time of their hospitalization. Furthermore, we consulted a separate table that stored information about medical tests including the name of the test, the date it was conducted, and the results of the test (Table 6).

By combining these diverse sources of data, our objective was to create a holistic view of each patient’s medical journey and gain valuable insights into their conditions, treatment progress, and overall health. Table 7 provides a summary of the utilized database tables along with their respective rationales, aimed at enhancing the understanding of the material by the readers.

As shown in Table 7, we were presented with a considerable volume of data generated by a real hospital. This untapped data reserve possessed the inherent potential to significantly enhance physicians’ performance and patient care. By utilizing advanced analytical techniques, these surplus data could be transformed into valuable insights, offering a wealth of information that can aid physicians in making more informed and accurate clinical decisions.

To ensure our analysis focused on the desired diseases, we began by refining the diagnosis table to include only the diagnoses that corresponded to our three target diseases. However, this process proved to be more complex than initially anticipated due to the hospital’s non-standardized data collection and generation practices. The diagnostic entries exhibited variations in formatting, including the use of abbreviations, mixed cases (uppercase and lowercase), and inconsistent naming conventions.

After applying our filtering criteria, all the selected entries underwent crucial validation and verification by our team’s physicians. Their thorough review provided an additional layer of scrutiny and assurance, enabling us to confidently proceed with our analysis.

Next, we proceeded to retrieve all the information about patients and admissions of patients who had been diagnosed with at least one of the remaining entries in the filtered diagnoses table. To ensure a holistic analysis, we extended our data-acquisition phase to encompass the real-time data. We narrowed down this table to include only the patients identified in the previous steps. For each disease, we began by merging all the information collected about patients and admissions, and the real-time data into a (python) Pandas DataFrame that we will now refer to as the “Hospitalization Dataset”. Subsequently, we created additional variables, including the patients’ age, duration of admission in days, and time of admission in minutes at each observation of the real-time data collection.

Additionally, we retrieved medical-examination data from the LabTests table. Specifically, for all three diseases, we obtained patients’ exam results for Troponin and N-terminal prohormone of brain natriuretic peptide (NT-proBNP) levels and we joined that information with patients’ and admissions data for each one of the three diseases, resulting in a dataset that we will refer to as the “Medical Tests Dataset”. Troponin and NT-proBNOP are biological markers specific to cardiac lesion and/or strain. Troponin is a cardiac-specific protein released when myocardium cells are injured. NT-proBNP is a pro hormone released by the heart upon volume or pressure overload. Troponin is a marker of ischemia, so it correlates with ST deviation, while NT-proBNP is from heart failure congestion. After collecting all the medical test data related to NT-proBNP, we conducted an examination of the data within our CRISP-DM data preparation stage. We performed various procedures, such as removing duplicate entries and ensuring that the tests were conducted during the patient’s hospitalization period. Additionally, we eliminated tests with implausible results, such as values of 0 or negative values. Despite these efforts, upon analyzing the data for each disease, we regret to report that the number of valid tests remained extremely low and insignificant for us to proceed with further analysis. As a result, the Medical Tests Dataset only included Troponin Tests and their corresponding results for each disease. The NT-proBNP data set did not have enough data to perform a valid analysis.

Overall, we began our individual analyses with two distinct datasets for each disease. The first dataset, referred to as “Hospitalization Dataset”, incorporated information about patients, admissions and real-time data measured throughout their hospitalization. The second dataset, named “Medical Tests Dataset”, comprised patient, admission, and test result information for Troponin. As our data preparation (acquisition, cleaning and filtering) phase was finally completed, we proceeded to better study and understand each disease.

### 4.1. Myocardial Infarction

#### 4.1.1. Hospitalization Dataset

Our EDA for Myocardial Infarction included a dataset of 260 patients. Among them, 57 patients were female, and 203 patients were male. This dataset consisted of 368,285 observations, with each observation representing a real-time data collection record for an individual patient. The age range of patients diagnosed with Myocardial Infarction varied from 16 years (the youngest patient) to 88 years (the oldest patient). In terms of data collection, we observed that one patient had the highest number of real-time data collection records, with a total exceeding 23,000. This patient was hospitalized for approximately two and a half months.

Then, we performed common data preparation procedures, such as removing duplicate entries and addressing missing values. After performing the aforementioned procedures, we proceeded to analyze certain parameters such as Heart Rate and Respiratory Rate, as they are the vital signs usually collect for this disease, since their variation can determine Ischemia.

**Figure 2:**
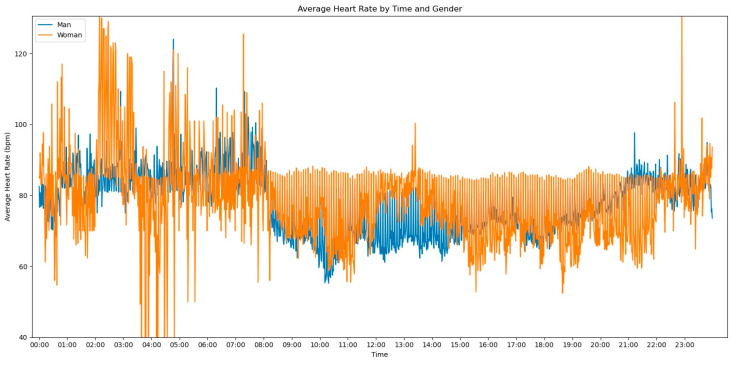
Average Heart Rate by time and gender.

In our initial descriptive statistics approach, we grouped the heart rate measurements by extracting the hourly pattern of each measurement. Our objective was to investigate whether the time of day had any influence on the frequency of heart rate readings. To further enrich the graph’s information, we also incorporated the gender variable to assess any significant differences, as shown in Figure 2.

**Figure 3:**
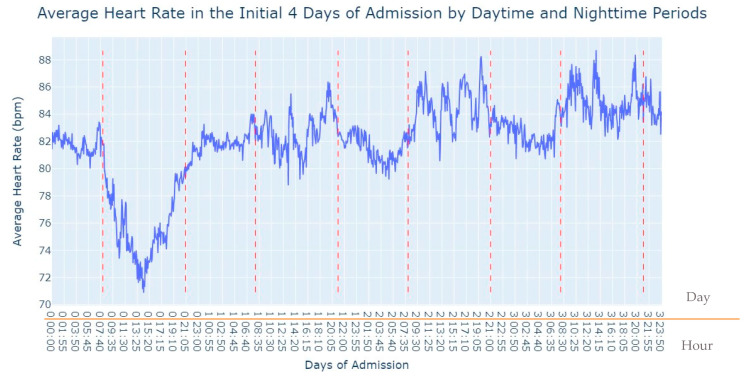
Average Heart Rate in the initial 4 days of admission by daytime and nighttime period.

Observing Figure 2, several notable patterns emerge. Firstly, it is evident that women tend to have higher rates of tachycardia compared to men. Additionally, an intriguing observation is that the average heart rate appears to be higher during the nighttime period compared to the daytime period. This fact prompted us to expand our analysis by incorporating the day of admission as an additional grouping factor for heart rate measurements. By including this level of grouping alongside the hour and minute of each measurement, our aim was to explore potential trends or variations in heart rate patterns throughout the duration of the patients’ admission days, as depicted in Figure 3.

**Figure 4:**
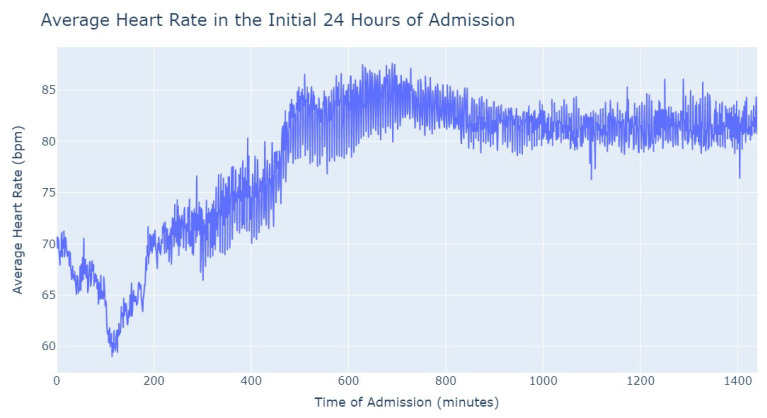
Average Heart Rate in the initial 24 h of admission.

As we can see in Figure 3, during the first 24 h of admission into the hospital, there is a decline in the patients’ average heart rate values during the daytime period. From 8 am until lunchtime, the values progressively decrease, and from lunchtime until the start of the nighttime period, they increase. In the subsequent days of admission, the average value oscillates between the 80s bpm during both the day and nighttime, with no noticeable differences. So, that phenomenon in the first 24 h of admission lead us to another analysis where we explored with more detail the evolution of heart rate during that period of admission. To accomplish this, we utilized the average values based on minutes of admission, focusing specifically on the time span from minute 1 to minute 1440, which corresponds to the first 24 h of admission. This analysis allowed us to explore how the heart rate changes over this crucial initial period of hospitalization, as seen in Figure 4.

It’s notable to observe in Figure 4 that the average heart rate progressively decreases until around 2 h after admission starts; this happens since patients with a Myocardial Infarction undergo angioplasty in the first hour.

This marks the lowest average heart rate, after which it begins to increase progressively, eventually stabilizing between 80 and 85 after 8 h of admission. This behavior of the average heart rate could possibly be influenced by medication or medical procedures (such as percutaneous coronary intervention), and once their effects take place, the value tends to stabilize.

**Figure 5:**
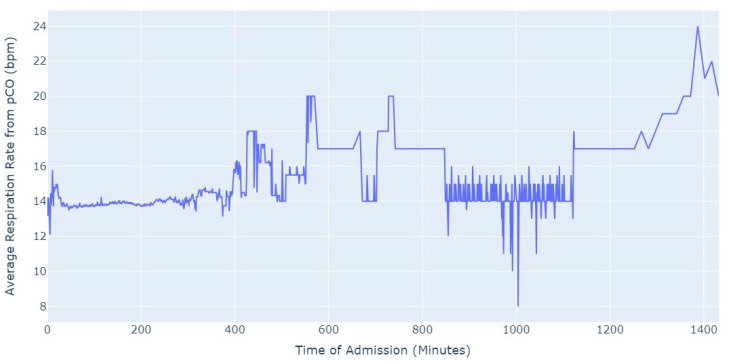
Average Respiration Rate for first 24 h of admission.

Shifting our focus, another measure chosen to evaluate patients’ conditions was the Respiration Rate (taken from thoracic impedance from EKG). Initially, we had more than 47,000 observations with this measure recorded. However, after ensuring that the value was greater than 0, we were left with only about 39,000 records. Out of these, 36,000 measurements were taken during the first 24 h of hospital admission. As a result, we focused our analysis on this subset of data, as depicted in Figure 5.

As we can see in Figure 5, the average value of the respiration rate during the first 24 h of admission (1440 min) oscillates between 12 and 20, with a decrease to 8 occurring close to 17 h after the admission start. Subsequently, there is an increase in the average value, which is observed close to the 24 h mark since the admission start.

#### 4.1.2. Medical Tests Dataset

Shifting our focus to the dataset of medical exams, we conducted an analysis to comprehend the progression of Troponin over time. Specifically, we examined 1546 observations of Troponin exams.

**Figure 6:**
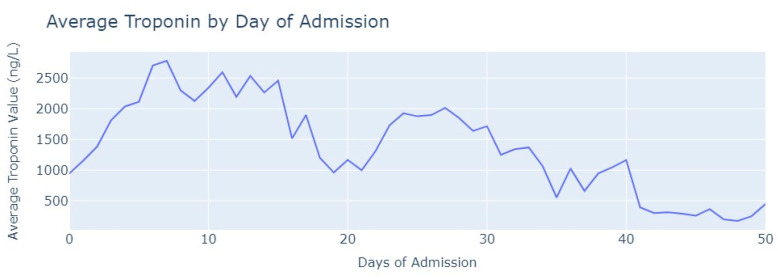
Average Troponin values by day of admission.

After removing duplicate entries and applying specific filters to ensure the inclusion of only relevant exams conducted during the hospitalization period, we successfully eliminated all tests that were not administered during the specified timeframe. As a result of these two procedures, our dataset was refined, consisting now of 1348 records for Troponin. To analyze the Troponin exams more effectively, we grouped them based on the average values per day of admission, Figure 6.

The average value of Troponin starts at 1000 ng/L and exhibits a tendency to increase during the initial days of admission, reaching a peak of more than 2500 ng/L on the 7th day, followed by an oscillating but progressively decreasing pattern. For healthy individuals, Troponin values are expected to be lower than 14 ng/L for healthy people, and it is evident that for patients diagnosed with myocardial infarction, these values never return to the considered normal range even after more than 1 month from the start of their hospital admission.

### 4.2. Pulmonary Thromboembolism

#### 4.2.1. Hospitalization Dataset

For the pulmonary thromboembolism disease, we had a total of 48 patients, consisting of 28 males and 20 females. The age range of the patients spanned from 0 years to 91 years, with the longest hospitalization duration lasting for 322 days. The dataset with real-time data consisted of 87,760 observations, with each row representing a real-time data collection instance for an individual patient.

As in the previous disease, we handled duplicates by removing them, and any instances of missing values were addressed by exclusion, ensuring the data’s integrity remained intact.

**Figure 7:**
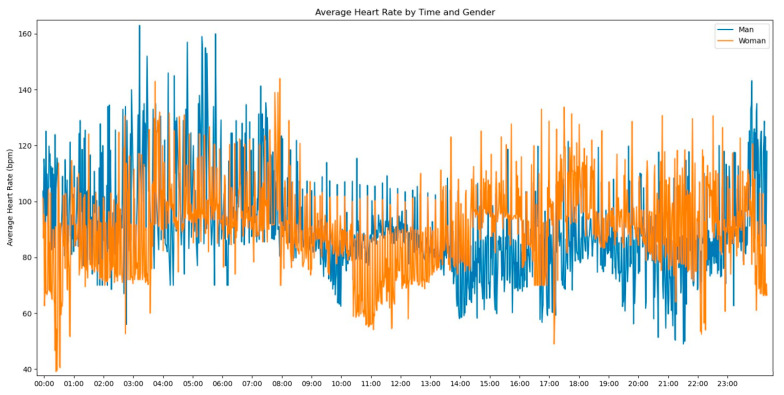
Average Heart Rate by time and gender.

Furthermore, we conducted an analysis of heart rate and respiration rate measures to identify patterns that could assist physicians in understanding the evolution of these parameters. The objective was to provide valuable insights into how these vital signs change over time and enable healthcare professionals to take appropriate actions based on a patient’s individual evolution compared to the typical behavior observed in the majority of patients. Once again, we commenced our analysis by examining heart rate patterns across hours and genders, with the aim of identifying intriguing trends, as shown Figure 7.

Figure 7 shows a distinction from myocardial infarction. The average Heart Rate values tend to be tachycardic, and there is no significant difference between men and women, even during the daytime or nighttime periods. The values oscillate between 60 bpm and 140 bpm, with men having a few average heart rate values above 140 bpm during the nighttime period.

**Figure 8:**
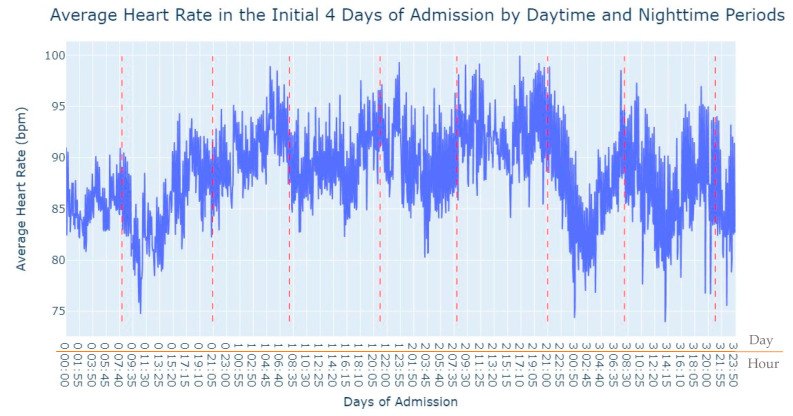
Average Heart Rate in the initial 4 days of admission by daytime and nighttime periods.

Then, we proceed our analysis by grouping Heart Rate values based on their day of admission and the specific hour and minute, computing the average value for each group, as seen in Figure 8. Subsequently, we plotted the resulting graph to examine any discernible patterns. Our primary objective was to investigate whether a similar pattern, as observed in Myocardial Infarction cases, would emerge. Specifically, we were interested in determining whether there was a notable minimum average heart rate during the daytime period of day 0, which could be indicative of a common trend in both conditions.

As depicted in Figure 8, the behavior of Heart Rate in this disease does not exhibit similarities with Myocardial Infarction. The values, rather, indicate signs of tachycardia, but there is no significant discernible pattern observed during different days of admission.

**Figure 9:**
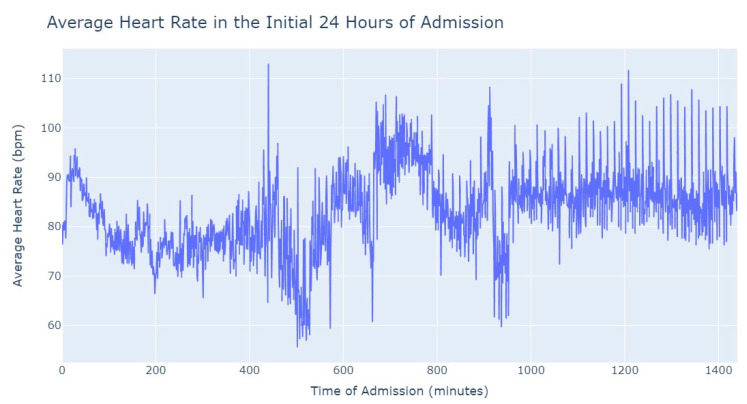
Average Heart Rate in the initial 24 h of admission.

Additionally, we conducted an analysis of the first 24 h of admission to investigate whether there were any instances of minimum or decreasing average heart rate values, Figure 9. The purpose was to ascertain whether such occurrences could be attributed to specific medical procedures or medication administered to the patient upon admission to the hospital.

In Figure 9, we can observe the same trend shown in Figure 8. The average heart rate values of patients diagnosed with pulmonary thromboembolism exhibit significant fluctuations. Patients frequently experience tachycardia, where heart rate values during the first 24 h can oscillate widely, ranging from under 60 to over 110 beats per minute (bpm).

**Figure 10:**
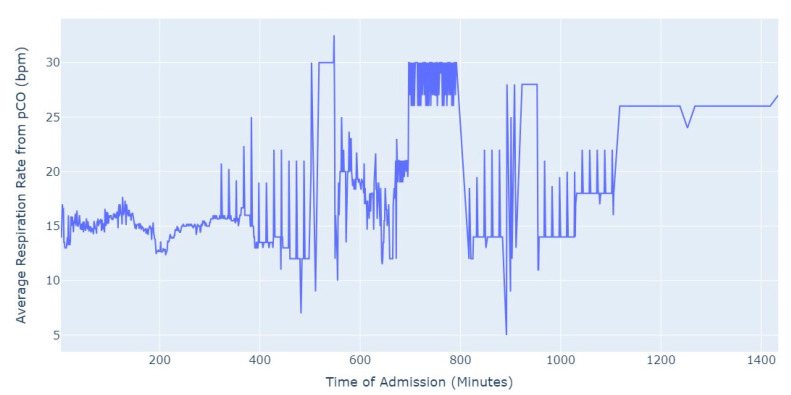
Average Respiration Rate for first 24 h of admission.

Shifting our focus to the Respiration Rate, we had more than 6500 valid observations. To ensure data integrity, we performed certain procedures, such as removing observations where the value of the respiration rate was equal to or lower than 0 (impossible values in this context). By applying these data cleaning procedures, we aimed to maintain the accuracy and reliability of the dataset for further analysis. Figure 10 presents the average respiration rate from EKG in the first 24 h of admission.

After approximately 5 h of hospitalization, the average value of respiration rate starts to exhibit significant fluctuations. During the initial 5 h, the average value remains relatively stable, around 15. However, after this period, the average respiration rate shows oscillations, varying between 5 and 30 in certain instances. This observation suggests that the respiration rate tends to become more erratic as the time since admission progresses.

#### 4.2.2. Medical Tests Dataset

**Figure 11:**
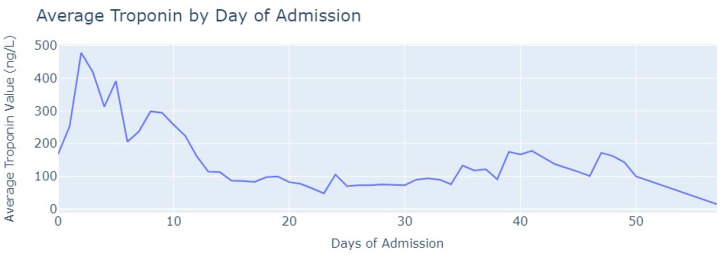
Average Troponin values by day of admission.

In terms of medical tests, we conducted a review to ensure we had a significant number of Troponin tests performed on patients diagnosed with pulmonary thromboembolism. The same procedures described in myocardial infarction were applied to ensure we had only relevant and valid tests and after careful examination, we aimed to retain 219 tests out of the total 244. Then, we grouped Troponin values based on the day of admission when the examination was conducted, and calculated the average value for each day, which is shown in Figure 11.

As observed in Figure 11, the average value of troponin initially increases, similarly to what is seen in myocardial infarction cases. However, after two days of admission, it starts to decrease with occasional minor increases. Notably, the highest average value of Troponin recorded was slightly below 500 ng/L, which contrasts with myocardial infarction cases where values reached 2500 ng/L.

### 4.3. Aortic Stenosis

#### 4.3.1. Hospitalization Dataset

As with the previous diseases, we began by presenting some descriptive statistics of the data under study. The dataset comprised 794,694 observations of real-time data collected from 660 patients, where 370 were male and 290 female. The ages of the patients ranged from 0 years to 93 years. Notably, the patient with the longest hospitalization period was admitted for 925 days, from 9 February 2017 to 23 August 2019.

After applying the same procedures as before, we conducted an analysis of the heart rate and respiration rate signals.

**Figure 12:**
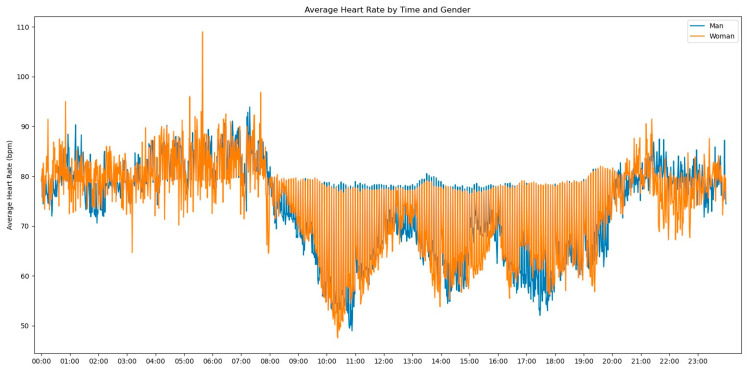
Average Heart Rate by time and gender.

As this disease had a substantial number of patients, with a relatively even distribution among genders, we first examined the average heart rate based on the hour and minute of measurement, as well as considering the patients’ gender; see Figure 12.

We can notice almost no difference between the genders in terms of the average heart rate values. The analysis indicates that both male and female patients with this disease exhibit similar trends in their heart rate patterns. It is also intriguing to observe a pattern that was previously noted in myocardial infarction but is not as prominent in pulmonary thromboembolism. The average heart rate values tend to be lower during the daytime period compared to the nighttime period, where the highest value from the daytime period almost corresponds to the lowest average heart rate value from the nighttime period.

**Figure 13:**
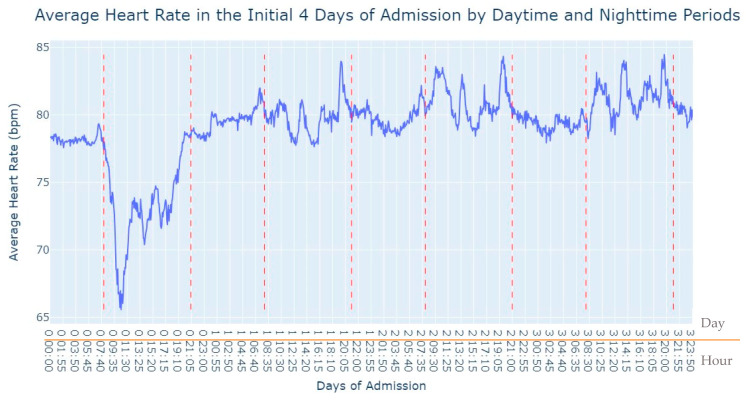
Average Heart Rate in the initial 4 days of admission by daytime and nighttime periods.

This fact led us to investigate the influence of the daytime period on the average heart rate values, the results of which are shown in Figure 13. By analyzing the heart rate data during daytime periods, we sought to discern potential patterns or variations that could shed light on how this specific time of day may impact the average heart rate in patients.

**Figure 14:**
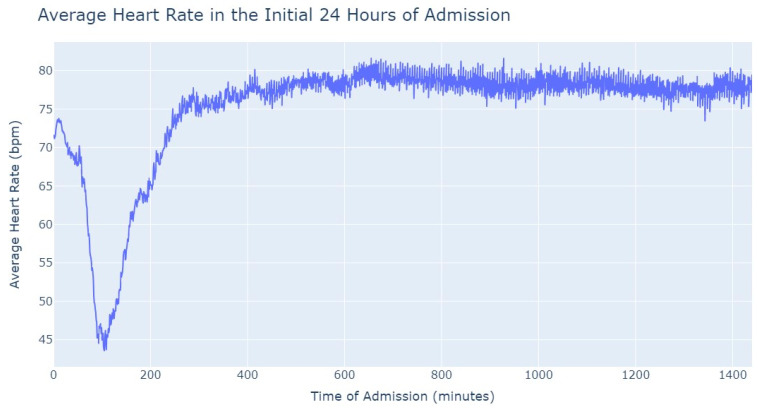
Average Heart Rate in the initial 24 h of admission.

Aortic stenosis exhibits a similar behavior to myocardial infarction. Those suffering from myocardial infarction are submitted to Angioplasty while patients suffering from aortic stenosis are admitted and in the next day have a scheduled procedure Transcutaneous Aortic Valve Implantation (TAVI) with sedation and or anesthesia. On the first day of admission (day 0), during the daytime period, the lowest average value of their heart rate is noted. From 8 am onwards, their heart rate starts decreasing (when they are submitted to TAVI), and throughout the daytime period, it never reaches the average value of heart rate seen during the nighttime period on the first day of admission. Subsequently, in the following days, the heart rate values show greater stability, both within each day and between the daytime and nighttime periods. Even in comparison with pulmonary thromboembolism, most of the data for aortic stenosis exhibits remarkable stability, except for the observed phenomenon on the first day of admission. The heart rate values demonstrate a consistent pattern over time, indicating relatively steady and consistent behavior in most cases. Our efforts were then focused on studying the first 24 h of admission, as depicted in Figure 14, to understand if it showed a similar behavior to myocardial infarction, where the average heart rate decreases and reaches its lowest value approximately one and a half hours after the admission starts.

Once again, Figure 14 presents a behavior quite like myocardial infarction in aortic stenosis patients. The average heart rate starts decreasing after admission and reaches its lowest value at approximately 2 h. Subsequently, the heart rate gradually increases until it reaches around 75 bpm. Over the remainder of the first 24 h, the average heart rate remains stable, fluctuating between 75 and 80 bpm. This consistent and characteristic pattern of heart rate changes in the initial 24 h of admission resembles the behavior typically seen in myocardial infarction cases. The behavior of heart rate in aortic stenosis and myocardial infarction is quite similar when compared to pulmonary thromboembolism, where tachycardia is more prevalent globally. However, unlike in the former cases, there is not a significant gradual decrease or increase in heart rate values.

**Figure 15:**
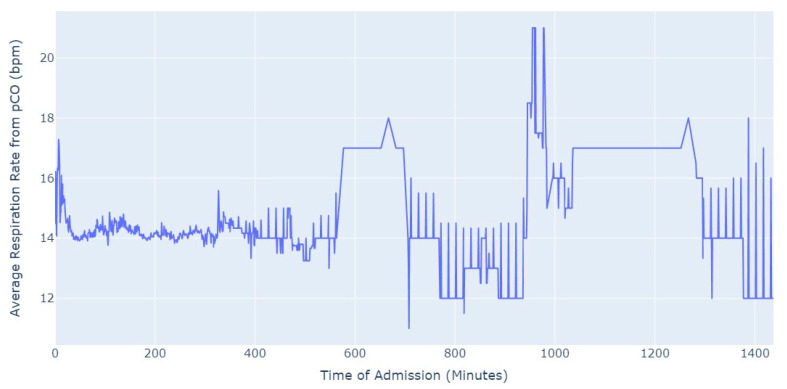
Average Respiration Rate for first 24 h of admission.

Shifting our focus to the Respiration Rate, we analyzed the values from the first 24 h of admission, Figure 15. Out of the total 63,000 observations, a substantial majority of 57,000 observations were specifically from the first 24 h of admission.

Once again, we observed new evidence of a behavior similar to myocardial infarction in aortic stenosis patients. The average values of Respiration Rate rarely exceeded the limits considered normal for healthy individuals, which typically fall within the range of 12 to 20 breaths per minute. Pulmonary thromboembolism did not exhibit a markedly different behavior, but it did surpass the anticipated values on multiple occasions.

#### 4.3.2. Medical Tests Dataset

**Figure 16:**
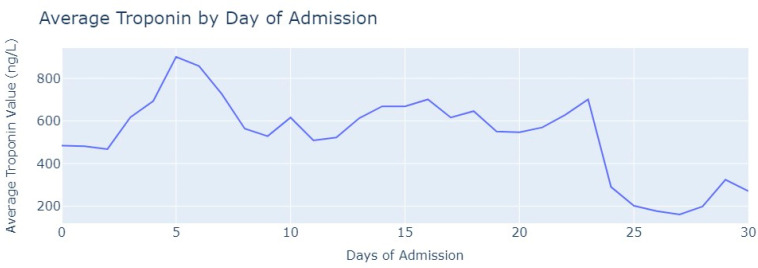
Average Troponin value by day of admission.

Analyzing the Troponin tests, we first filtered these exams to ensure they were conducted within the hospitalization period. Out of the total Troponin tests, 2872 exams were considered valid and were included in the analysis. Then, we followed a similar approach to before by grouping the values of tests based on the admission day when they were conducted. Subsequently, we calculated the average value of Troponin for each day, as seen in Figure 16.

Conversely, the values of Troponin in aortic stenosis patients across the day of admission are not similar to Troponin figures in myocardial infarction patients, contrasting in their heart rate and respiration rate. In aortic stenosis, the average Troponin value reaches its highest point after 5 days of admission and then gradually decreases but remains relatively stable until the 24th day of admission. Unlike myocardial infarction, the Troponin values in aortic stenosis patients never reach levels as high as 1000 ng/L. Even when compared with pulmonary thromboembolism, the behavior of troponin differs. In aortic stenosis cases, the highest value is only reached after 5 days, whereas in pulmonary thromboembolism, it occurs earlier. By the 12th day, troponin levels in pulmonary thromboembolism drop below 200 ng/L, whereas in aortic stenosis, this value is only achieved after 25 days of admission. The distinct behavior of Troponin in aortic stenosis patients is noticeably different.

## 5. Machine Learning Predictive Models—Myocardial Ischemia Prediction

Moving beyond the Exploratory Data Analysis, our focus shifted to the machine learning (ML) modeling phase, where our aim was to predict myocardial ischemia. This predictive capability allows us to anticipate critical events and provide physicians with the tools necessary for early interventions. The overarching purpose of our data analysis was to aid physicians in making well-informed and accurate decisions, ultimately enhancing patient outcomes, and elevating the overall quality of care.

To initiate our predictions, we employed the Hospitalization Dataset for each of the three diseases. This dataset includes variables known as ST Segment Lead that are represented as float values, which can be either positive or negative. Myocardial ischemia can be detected using ST segment modification, this being an established marker of cardiac injury without cellular death. It may happen as part of an unfavorable evolution of the disease or therapeutic insufficiency. We evaluated these values using the ST-segment-T wave criteria [25], which is suggestive of myocardial ischemia (MI). The criteria for ST-elevation and ST-depression are distinct, and for each variable under study, specific rules were applied to determine the presence of myocardial ischemia. For each disease, we analyzed each record and verified whether, according to the values of our ST-Segment variables and the aforementioned criteria, myocardial ischemia was present or not. A Boolean variable was created to represent the phenomena. Table 8 presents the number of observations (rows) in the Hospitalization Dataset, the number of diagnosed patients, the number of patients that had MI and the number of observations with myocardial ischemia for each disease under study. Please note that these patients were admitted to the hospital with cardiac diseases and were measured with a non-regular frequency of 1–5 min, which could explain the higher number of cases of myocardial ischemia.

Starting our predictions, the initial idea was to utilize shift variables to attempt a prediction of whether a patient would experience myocardial ischemia in the future. For each ST variable, the Heart Rate variable and the MI variable, we selected the past values (lag) based on the autocorrelation of the patient with the most observations. Since the dataset contained multiple records for various patients, it was crucial to check the autocorrelation for only one patient to ensure accurate predictions of myocardial ischemia based on shift values for that specific patient. We performed this procedure for each one of the three diseases under study, utilizing the respective Hospitalization Dataset. Autocorrelation, in essence, quantifies the extent of similarity between a variable and its past values across various time intervals. The interdependence over time within the dataset can substantially impact the performance of a model. A noticeable autocorrelation could suggest a strong temporal relationship between ST Segment measurements or MI occurrences at different points in time. This indicates that the current ST Segment value could potentially be influenced by its own historical values. In the domain of predictive modeling, autocorrelation plays a pivotal role in refining the accuracy of predictions. This phenomenon enables us to utilize past values effectively to formulate forecasts for the future.

**Figure 17:**
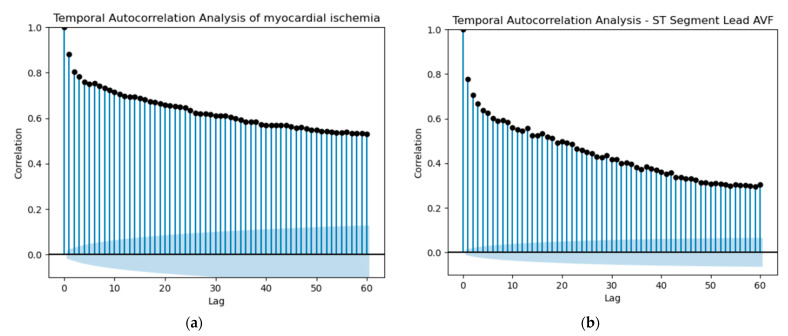
Here we represent as an example the autocorrelation plots for the variables myocardial ischemia and ST Segment Lead AVF, where (**a**) represents the autocorrelation for myocardial ischemia and (**b**) represents the autocorrelation for ST segment Lead AVF.

Based on the observations in Figure 17 and the autocorrelation graphs for all ST Segment variables, Heart Rate and MI, we selected a lag of 13 and created the respective shifted variables. It is important to note that lag 13 could represent 13 min or more, considering the irregular frequency of data collection, which could range from 1 to 5 min.

Categorical variables, such as ethnicity, sex, or blood group, were converted into dummy variables. Afterward, we calculated the correlation between each variable and our target variable, myocardial ischemia, and selected the ones with the highest correlation, as shown in Table 9. Of these selected variables, Troponin was discarded since it has no predictive value; as troponin is an indicator of cellular necrosis, it is not included in the analysis of ischemia (since it only appears a posteriori).

In addition to this selection, we also included PatientDboid (the primary key for the patients DB table), to conduct further analysis. We also combined all the data from the hospitalization datasets for the three diseases to create a global model. The same procedures were conducted as with each individual disease.

For each disease, after selecting the variables to include in the model, we decided to split the data between train and test sets based on patients. We randomly selected one patient within the top five with the most rows for each disease and used them for the test set, while excluding them from the training set. Afterward, we dropped the PatientDboid column and created separate sets of X and Y for both the training and test datasets. For the global model, we randomly split the data into training and test sets using an 80–20 proportion. The algorithms we employed for the machine learning modeling were Random Forest, Naïve Bayes, and Neural Network. We constructed a Neural Network with two hidden layers, each consisting of 1024 and 512 neurons, respectively. The activation function used in both hidden layers was ReLU (Rectified Linear Unit), while the output layer employed the Sigmoid activation function. For optimization, we utilized the Adam optimizer and the binary cross-entropy loss function.

Table 10 presents the results, highlighting the best-performing algorithm for each disease. We evaluated the performance of each algorithm using all four standard classification evaluation metrics (F1-Score, Accuracy, Precision, Recall). Additionally, the table includes the number of records in the training and test sets for each disease.

As evident from the results in Table 10, the best-performing algorithms achieved impressive scores close to 90% for each evaluation metric, indicating their good predictive capabilities in identifying myocardial ischemia with a time lag of 13. These highly promising outcomes offer strong encouragement for the subsequent stages of implementation, as the algorithms demonstrated the potential to assist physicians in real time in providing timely advice regarding myocardial ischemia occurrence and forecast in patients.

When comparing the global model with the disease-specific models, the results are not as impressive, if we think of the first case. However, the global model is more versatile as it is not solely trained on data from one disease. It is also important to mention that each model can be applied to patients with any disease, with the only requirement being the data specified in Table 9 for the chosen model. These innovative results underscore the significance of further steps in refining and deploying the algorithms in clinical settings. The potential benefits of such predictive models are immense, as they can aid healthcare professionals in proactively managing, forecasting and offering personalized care to patients at risk of myocardial ischemia.

## 6. Discussion

In this section, we engage in a comprehensive discussion of the findings and implications of our study, while also acknowledging the limitations and ethical considerations inherent in AI-driven healthcare research.

In the context of our research question, “how can fusion of Exploratory Data Analysis (EDA) techniques and predictive machine learning models assist medical staff in accurate clinical decision-making and facilitate timely medical interventions of a preventive nature?”, we presented charts for our use cases in three specific diseases—acute myocardial infarction, pulmonary thromboembolism and aortic stenosis—that represent a range of studied CVDs. We employed machine algorithms to predict MI within a 13 min window, for patients diagnosed with these studied diseases. The implementation of a global model with all the patients (without filtering by disease), where we achieved 86% for each evaluation metric, demonstrates its ability to generalize. The best result for predicting MI was achieved when trained and evaluated for patients suffering from Pulmonary Thromboembolism and Aortic Stenosis. Our ability to forecast myocardial ischemia incidents with these levels of accuracy, particularly within a 13 min window, holds promising implications for timely medical interventions and improved patient outcomes.

As with any research endeavor, it is essential to acknowledge the limitations of our study. We confronted the challenges posed by the lack of standardization in data collection procedures and the prevalence of unstructured clinical information in Electronic Medical Records. These limitations impacted the accuracy and generalizability of our predictive models, and we recognize the need for ongoing efforts to enhance data quality and standardization.

The use of AI in healthcare necessitates a robust consideration of ethical implications. We acknowledge our access to the clinical data of patients admitted to the Intensive Care Units of Hospital de Santa Maria, in the framework of FCT project DSAIPA/AI/0122/2020 AIMHealth, and the work [10], where DSAs were signed and homomorphic encryption was implemented. These ethical safeguards protected patient privacy and confidentiality while enabling critical research.

In the spirit of continuous improvement, we will engage in self-critique by identifying areas for enhancement and future research directions. Our exploration of myocardial ischemia prediction, while promising, remains a singular facet of AI applications in cardiovascular healthcare. We advocate for a broader exploration of AI’s potential in addressing various cardiovascular diseases, and on behalf of the study that was conducted [10], we will focus on further enhancing the robustness and generalizability of our predictive models by integrating data from multiple hospitals and medical institutions. This collaborative approach aims to encompass a broader patient population and provide a more comprehensive understanding of cardiovascular diseases.

## 7. Conclusions

Our exploratory data analysis of the three studied diseases enables physicians to grasp patterns in Heart Rate, Respiration Rate, and Troponin values. Going forward, they can compare data from new patients with the established behavioral norms derived from previous patients diagnosed with the same disease. We believe that this approach enables physicians to gain a more profound understanding of the recovery status and spend more time with patients that show different behaviors. For future work, we suggest conducting an analysis of additional medical tests, such as NT-proBNP.

In our study, it is also noteworthy to observe that aortic stenosis and myocardial infarction exhibit certain similarities, which stand in contrast to pulmonary thromboembolism. The most prominent evidence lies in Heart Rate, where in both diseases, the average value progressively decreases after admission, reaching its lowest point approximately 2 h after admission before beginning to rise again.

In this paper, we presented another valuable AI tool, which performed the prediction (forecast) of myocardial ischemia. Our literature review uncovered no relevant studies addressing the use of machine learning to assist physicians in evaluating the progression of patients’ conditions post-diagnosis, showing the relevance of this study. If physicians were alerted 13 min in advance that a patient might experience myocardial ischemia, with an accuracy of around 90%, they could take proactive measures rather than reactive ones, and we believe our AI modeling tool can lead them in that direction. Upon refinement, this model may be further tested prospectively to predict ischemia and arrhythmia in monitored cardiac patients. We would like to point out, in addition, that we have shown that our machine learning model can be applied to any other disease, with the sole requirement being that the patient must be connected to a medical device that collects ST Segment Variables.

It is also important to mention that applying exploratory data analysis to other diseases could provide a better understanding of their progression, but it must be performed within a singular analysis.

## Figures and Tables

**Table 1:** Topics found in literature review.

Topic	Reference	Number of Documents
Heart Disease Prediction	[12,13,14,15,16,17,18,19,20]	9
IoT	[12,13,14,15,16,21,22]	7
Risk Assessment	[16,17,18,19]	4
Big Data	[13,20]	2
Mortality Prediction	[19]	1
Recommender Systems	[23,24,25]	3
Clustering	[26]	1
Blockchain	[15]	1

**Table 2:** RT-Data DB table composition.

Variable Name	Variable Description
RTDATADBOID	Database Object ID for each collection of real-time data
CREATIONDATE	Date and time of real-time data collection
RESPIRATION RATE FROM EKG	Respiration Rate Value
ST SEGMENT LEAD V5	ST segment deviation from baseline in the ECG Leads V5
ST SEGMENT LEAD V4	ST segment deviation from baseline in the ECG Leads V4
ST SEGMENT LEAD V3	ST segment deviation from baseline in the ECG Leads V3
ST SEGMENT LEAD V2	ST segment deviation from baseline in the ECG Leads V2
ST SEGMENT LEAD V1	ST segment deviation from baseline in the ECG Leads V1
ST SEGMENT LEAD AVF	ST segment deviation from baseline in the ECG Leads aVF
ST SEGMENT LEAD AVR	ST segment deviation from baseline in the ECG Leads aVR
ST SEGMENT LEAD AVL	ST segment deviation from baseline in the ECG Leads aVL
ST SEGMENT LEAD III	ST segment deviation from baseline in the ECG Leads III
MEAN ARTERIAL PRESSURE 2	Mean Arterial Pressure Value
DIASTOLIC PRESSURE (ART.) 2	Diastolic Pressure Value
SYSTOLIC PRESSURE (ART.) 2	Systolic Pressure Value
HEART RATE	Heart Rate Value

**Table 3:** Diagnoses DB table composition.

Variable Name	Variable Description
DIAGDBOID	Database Object ID for each diagnosis
DIAGDESC	Description of each diagnosis
DIAGTYPEDESC	List of diagnoses
DIAGCODE	Code associated with a particular diagnosis in the list of diagnoses

**Table 4:** Patient DB table composition.

Variable Name	Variable Description
PATIENTDBOID	Database Object ID for each patient
BIRTHDATE	Patient birth date
BLOODGROUP	Patient blood group
SEX	Patient gender
ETHNICITY	Patient ethnicity

**Table 5:** Admission DB table composition.

Variable Name	Variable Description
ADMISSIONDBOID	Database Object ID for each admission
STARTED	Admission start datetime
ENDED	Admission end datetime
WEIGHT	Admission weight
HEIGHT	Admission height

**Table 6:** Laboratory test DB table composition.

Variable Name	Variable Description
LABTESTDBOID	Database Object ID for each Laboratory Test
STARTED	Datetime of test realization
ANALYSISDESC	Name of analysis component
VALUE	Result for analysis component

**Table 7:** Summary of utilized DB tables and their description.

DB Table	Description	No. Observations
Patients	Obtain personal information to build a patient profile	512,764
Admission	How long a patient was hospitalized	1,159,139
Diagnoses	Filter by desired diseases	126,126
LabTests	Extract date and result from specific exams	8,043,764
RT_Data	Real-time data monitored during patients’ hospitalization	30,404,477

**Table 8:** Presence of myocardial ischemia (MI) for each disease.

Disease	No. Observations	No. Patients	Patients’ w/MI	No. Observations w/MI
Myocardial Infarction	368,285	260	254	144,273
Pulmonary Thromboembolism	87,760	48	22	17,357
Aortic Stenosis	794,694	660	649	394,967

**Table 9:** Variables used in models by disease.

Myocardial Infarction	Pulmonary Thromboembolism	Aortic Stenosis	Global Model
Height	Height	Shift_Myocardial_Ischemia	Age
Age	Weight	Shift_Heart_Rate	Shift_Myocardial_Ischemia
Shift_Myocardial_Ischemia	Age	Shift_Segment_Lead_AVL	Shift_Segment_Lead_AVL
Shift_Segment_Lead_AVL	Shift_Myocardial_Ischemia	Shift_Segment_Lead_AVR	Shift_Segment_Lead_III
Shift_Segment_Lead_AVF	Shift_Heart_Rate	Myocardial_Ischemia	Ethnicity_Caucasian
Shift_Segment_Lead_III	Shift_Segment_Lead_AVL		Myocardial_Ischemia
Ethnicity_Caucasian	Shift_Segment_Lead_AVF		
Myocardial_Ischemia	Shift_Segment_Lead_III		
	Ethnicity_Caucasian		
	Sex_Female		
	Myocardial_Ischemia		

**Table 10:** Performance of algorithms by disease.

Disease	Algorithm	F1-Score	Accuracy	Precision	Recall
**Myocardial Infarction**	Random Forest	0.81	0.82	0.81	0.80
# Train Set: 356,905	Naive Bayes	0.81	0.82	0.81	0.81
# Test Set: 8023	Neural Network	0.86	0.82	0.86	0.86
**Pulmonary Thromboembolism**	Random Forest	0.92	0.94	0.92	0.91
# Train Set: 80,685	Naive Bayes	0.73	0.75	0.73	0.82
# Test Set: 6595	Neural Network	0.87	0.94	0.87	0.87
**Aortic Stenosis**	Random Forest	0.83	0.87	0.84	0.83
# Train Set: 778,744	Naive Bayes	0.83	0.86	0.83	0.83
# Test Set: 9365	Neural Network	0.91	0.87	0.91	0.92
**Global Model**	Random Forest	0.86	0.86	0.86	0.86
# Train Set: 990,756	Naïve Bayes	0.84	0.84	0.84	0.84
# Train Set: 247,690	Neural Network	0.84	0.85	0.84	0.83

## Data Availability

Not applicable.
